# Premature Spinal Bone Loss in Women Living with HIV is Associated with Shorter Leukocyte Telomere Length

**DOI:** 10.3390/ijerph15051018

**Published:** 2018-05-18

**Authors:** Shirin Kalyan, Neora Pick, Alice Mai, Melanie C. M. Murray, Kristen Kidson, Jackson Chu, Arianne Y. K. Albert, Hélène C. F. Côté, Evelyn J. Maan, Azita Goshtasebi, Deborah M. Money, Jerilynn C. Prior

**Affiliations:** 1 Department of Medicine, University of British Columbia, Vancouver, BC V5Z 1M9 Canada; shirin.kalyan@ubc.ca (S.K.); alice.y.mai@gmail.com (A.M.); kmsokalski@gmail.com (K.K.); cts.jackson@gmail.com (J.C.); jerilynn.prior@ubc.ca (J.C.P.); 2 Division of Endocrinology; Centre for Menstrual Cycle and Ovulation Research, BC Centre for the Canadian Multicentre Osteoporosis Study, University of British Columbia, Vancouver, BC V5Z 1M9, Canada; azita.goshtasebi@ubc.ca; 3 Oak Tree Clinic, British Columbia Women’s Hospital, Vancouver, BC, V6H 3N1, Canada; Melanie.murray@cw.bc.ca (M.C.M.M.); emaan@cw.bc.ca (E.J.M.); deborah.money@ubc.ca (D.M.M.); 4 Department of Medicine, Division of Infectious Disease, University of British Columbia, Vancouver, BC V6Z 1Y6, Canada; 5 BC Women’s Health Research Institute, British Columbia Women’s Hospital, Vancouver, BC V6H 3N1, Canada; Arianne.albert@cw.bc.ca; 6 Department of Pathology & Laboratory Medicine, University of British Columbia, Vancouver, BC V6T 2B5, Canada; hcote@pathology.ubc.ca; 7 Department of Obstetrics and Gynecology, University of British Columbia, Vancouver, BC V6Z 2K8, Canada

**Keywords:** HIV infection, osteoporosis, women, antiretroviral therapy, leukocyte telomere length, bone mineral density

## Abstract

With advances in combination antiretroviral therapy (cART), people living with HIV are now surviving to experience aging. Evidence suggests that individuals living with HIV are at greater risk for low bone mineral density (BMD), osteoporosis, and fractures. Better understanding of the pathophysiology of bone health in women living with HIV (WLWH) is important for treatment strategies. The goal of this study was to explore new biological factors linked to low BMD in WLWH. Standardized BMD measures of WLWH were compared to reference values from an unselected population of women from the same geographical region of the same age range. Linear regression analysis was used to assess relationships among health-related characteristics, cellular aging (measured by leukocyte telomere length; LTL), cART, and BMD of WLWH. WLWH (*n* = 73; mean age 43 ± 9 years) had lower BMD *Z*-scores at the lumbar spine (LS) (mean difference = −0.39, *p* < 0.001) and total hip (TH) (−0.29, *p* = 0.012) relative to controls (*n* = 290). WLWH between 50 and 60 years (*n* = 17) had lower *Z*-scores at the LS (*p* = 0.008) and TH (*p* = 0.027) compared to controls (*n* = 167). Among WLWH, LS BMD was significantly associated with LTL (R^2^ = 0.09, *p* = 0.009) and BMI (R^2^ = 0.06, *p* = 0.042). Spinal BMD was adversely affected in WLWH. Reduction of LTL was strongly associated with lower BMD and may relate to its pathophysiology and premature aging in WLWH.

## 1. Introduction

With the progress in HIV combination antiretroviral therapy (cART) and the aging of people living with HIV (PLWH), there is increasing evidence that living with HIV is a significant risk factor for low bone mineral density (BMD) and fragility fractures [1,2,3,4,5,6,7,8]. A meta-analysis of 20 studies in PWLH compared to uninfected controls [9] showed the pooled odds ratios (ORs) for having osteopenia and osteoporosis were 6.4 and 3.7, respectively. A previous case-control analysis of Canadian women living with HIV (WLWH) (mean age of 38 years) and age-matched controls derived from the national Canadian Multicentre Osteoporosis Study (CaM*os*) showed WLWH had an OR of 1.7 for having experienced a prevalent fragility fracture (i.e., low trauma fracture, such as with a fall from standing height) [6]. Similarly, a Spanish population-based study including both men and women reported that an HIV diagnosis increased the risk of a hip fracture by 5-fold, independent of sex, age, tobacco or alcohol use, and comorbidities [10]. Of note, despite osteoporosis and incident fractures being more common in menopausal women in the general population [11], studies of bone disease in PLWH have primarily been skewed towards men [12,13]. Given that >50% of PLWH globally are women, with the majority being of reproductive age [14], a greater understanding of the pathophysiology of bone health in WLWH is needed to develop optimal preventative and treatment strategies as they age.

Reasons for this increased prevalence of bone fragility in PLWH are multifactorial and complex. Aside from the direct effect of the HIV virus on immune homeostasis and its potential contribution to bone loss [7,8,15], PLWH also often have higher prevalence of risk factors for low BMD and fractures, which include poverty and poor nutrition, higher rates of drug use (including illicit and intravenous drugs, tobacco, and alcohol), lower body weight, lower vitamin D levels, and higher risk for hypogonadism (ovulatory and cycle disturbances in women and lower testosterone levels in men) [3,7,16,17]. In addition to these factors, initiation of cART is associated with a short-term (<2 year) accelerated bone loss of between 2% and 6% that varies with the type of cART [18].

It is also increasingly evident that both HIV infection and cART exposure are associated with accelerated aging, a process described as the declining ability of an organism to resist stress, damage, and disease [19]. Mitochondrial aging and telomere shortening are the basis of two widely accepted theories of aging [20,21]. HIV infection induces inflammation, causing oxidative stress that can damage both telomeres and mitochondrial DNA (mtDNA) [15]. Treatments, including nucleoside reverse transcriptase inhibitors (NRTI), can induce mtDNA alterations [22] and may accelerate telomere loss [23], a phenomenon that has been linked with age-associated diseases, such as osteoporosis, in some [24], but not all, studies [25]. Leukocyte telomere length (LTL) was reported to be associated with decreased BMD of the spine and forearm, but not of the femoral neck, in all 2150 women (aged 18–79) whose health administrative data were included in a whole population-based study in the United Kingdom [24]. Thus, the relationship between markers of accelerated aging and bone fragility requires further investigation in WLWH, since it may provide insight into the pathophysiology of metabolic bone disease in this at-risk group, and lead to interventions to reduce that risk.

In this cross-sectional study, we investigated the prevalence of abnormal BMD values in a cohort of WLWH in British Columbia (BC), Canada. We examined the relationships of BMD values with clinical and HIV-specific osteoporosis risk factors, and compared both younger and older (aged ≥50 years) WLWH’s data with a reference, population-based, age-appropriate cohort.

## 2. Materials and Methods

### 2.1. Study Design and Populations

This analysis is a cross-sectional comparison of data from an unselected local reference population (CaM*os*) compared with a subset of participants in the CARMA (Children and Women: AntiRetrovirals and Markers of Aging) study cohort at the Oak Tree Clinic, BC Women’s Hospital in Vancouver. CARMA began enrollment in December 2008 (UBC REB #H08-02018): its objective was to identify how HIV infection, cART use, and other factors relate to cellular aging in women and children living with/affected by HIV. At the time this substudy was initiated, there were 239 PLWH (192 women, 45 men, and 2 transgendered persons) enrolled in CARMA. At study enrollment and at annual or biennial visits thereafter, demographic and clinical information were obtained through a combination of self-reported data and medical chart review. This included participants’ general and HIV-specific medical histories, hepatitis C status (HCV) status, menstrual cycle history, cART history, smoking and alcohol history, and current substance/drug use. For the latter, a composite variable was created as described before [26]. Briefly, drugs (alone or in combination), including cocaine, heroin, crack, methamphetamines, marijuana, and methadone were classified on the basis of frequency (i.e., daily, weekly, monthly, or less) and the variable, “drug users (daily–weekly) yes/no” was created whereby subjects (regardless of duration of use) who used these substances on a weekly to daily basis were categorized as drug users. Those who never used drugs, experimented with drugs a few times in their lifetime, or rarely used drugs were categorized as “not drug users”. The coding into one category or the other was by a single clinician. Participants also provided a blood sample at each visit for analysis, which included LTL. The study was conducted in accordance with the Helsinki Declaration and received ethics approval through the University of British Columbia (ethics number: H08-02018).

The BMD control (reference) population consisted of women participants in the Canadian Multicentre Osteoporosis Study (CaM*os*) from British Columbia (BC) who were between the ages of 25 and 60. CaM*os* is a longitudinal study investigating factors relating to the bone health of Canadians, and participants were randomly selected based on residential addresses within a 50 km radius of each of the nine centers across Canada, as previously described [27]. We used the local CaM*os* participants as BMD comparison controls, as it is considered to be more appropriate to standardize BMD to the population from which subjects are derived, as opposed to the National Health and Nutrition Examination Survey (NHANES) [27], and geographic variation is an important factor in fracture risk [28].

Bone health outcome measures had not been specifically collected before 2013 in CARMA. Dual energy X-ray absorptiometry (DXA) became part of routine clinical care since this clinic published the first paper on fracture risk in WLWH in Canada [6]. We performed a retrospective chart review to obtain DXA data for the 192 WLWH participants (≥19 years of age). All WLWH who had undergone a BMD measurement by DXA as part of their clinical care, within 6 months of their CARMA visit and data collection, were included in this analysis; 73 WLWH met these inclusion criteria. Since we lacked complete objective data for reproductive status for all women, osteopenia and osteoporosis assessments were based on BMD *T*-Scores for women ≥50 years of age. For women <50 years of age, we calculated the standard deviation of age-matched population-based data (*Z*-scores) for those who were younger (detailed under bone measure assessments). Height and weight were measured (without shoes and wearing light clothing) before each DXA assessment; body mass index (BMI, weight in kg/height in meters squared) was calculated based on these measurements.

### 2.2. Areal Bone Mineral Density (BMD) Assessments

All BMD measurements were performed by DXA using a Hologic QDR 4500W instrument at BC Women’s Hospital, which was verified on the CaM*os* phantom [27] so that accurate BMD comparisons could be made to CaM*os* region-specific population-based data; the women from the CaM*os* reference cohort were also scanned on a Hologic instrument also validated against the CaM*os* phantom. Description of the referencing of all BMD data to the CaM*os* phantom is available from Berger et al. [29]. Measurements were taken at the lumbar spine (L1–4; LS), femoral neck (FN), and total hip (TH). To assess how the BMD of our cohort of WLWH compared to the general population, we calculated *Z*-scores (by decade of age) from the BMD values of an unselected cohort of women enrolled in the regional BC CaM*os* [27]. This enabled us to control for known geographic variation in BMD measures [30], and allowed a standardized age-adjusted method of comparing BMD in premenopausal and menopausal women’s data. *Z*-scores that are used in comparing BMD of a cohort or patient with a control population are generally age, sex, and ethnicity matched [31]. Since we could not match on ethnicity because of cohort differences, data for these younger women were compared based on age and sex. A normal *Z*-Score is defined as being >−2.0, while a *Z*-Score of ≤−2.0 is below the expected range for age [31]. For menopausal women or those ≥50 years, the World Health Organization (WHO) categorizes BMD as normal, osteopenic, or osteoporotic based on *T*-Scores (standard deviations in reference to the mean BMD of a healthy 25–29 year old, sex-matched reference population) [32]. Osteoporosis is defined as having a BMD *T*-score ≤ −2.5. Osteopenia is defined as a BMD with a *T*-Score between < −1.0 and −2.5. Normal bone is having a *T*-score ≥ −1.0. The majority of HIV-positive patients in the Oak Tree Clinic population reported taking calcium and vitamin D supplements, as recommended and frequently provided free of charge by their health care team.

### 2.3. Relative Average Leukocyte Telomere Length (LTL) Assay

Venous blood (0.1 mL) collected on the day of the closest CARMA visit and stored at −80 °C was extracted. Whole-blood relative mean LTL was measured by monoplex quantitative Polymerase Chain Reaction (qPCR), as described elsewhere [33].

### 2.4. Hepatitis C Virus (HCV) Infection Status

Hepatitis C virus (HCV) RNA testing was done on 200 µL of stored plasma, processed the same day as the blood used for LTL measurement. Briefly, HCV viral RNA was extracted from plasma using MagMAX™-96 Viral RNA Isolation Kit (Life Technologies, Carlsbad, CA, USA) following the manufacturer’s protocol. HCV RNA was detected by qPCR using probes and primers described elsewhere [34]. The cycling parameters for the ABI 7900HT Fast Real-Time PCR system were as follows: 1 cycle of 50 °C for 30 min, 1 cycle of 95 °C for 20 s, and 40 cycles of 95 °C for 1 s followed by 60 °C for 20 s. The limit of detection was approximately 100–200 IU/mL for genotypes 1, 2, 3, and 4.

### 2.6. Statistical Methods

The calculated BMD standard deviations (*Z*-scores) of the cohort of WLWH from CARMA were plotted using Tukey box-plots and assessed for deviation from BC CaM*os* controls. One sample t-test was used to determine the mean differences in BMD *Z*-scores at each bone measurement site and was reported with 95% confidence intervals [CI]. For women ≥50, we also compared BMD *T*-Scores of WLWH from CARMA and similarly aged BC CaM*os* women, as well as assessing the difference in the incidence of osteopenia and osteoporosis (BMD *T*-Score < −1.0) between the two cohorts using Fisher’s exact test. Variables used were assessed for Gaussian distribution using D’Agostino–Pearson omnibus normality test. Linear regression modeling was used to explore the relationship between variables of interest (age, BMI, parity, smoking, history of illicit drug use, active HCV co-infection, lifetime protease inhibitor (PI) use, lifetime tenofovir (TDF) use, current CD4 count, HIV plasma viral load, and LTL and BMD at the LS, TH, and FN). Univariately significant variables were added to a full model and tested for fit and collinearity by examining tolerance and the variance inflation factor. For comparison of contributions to the model, we used standardized β coefficients that are the equivalent of converting each variable into a *Z*-score so that it can be compared for its relative importance with each of the other variables in a regression model. We used SPSS version 23.0 (IBM, Armonk, New York, USA) for analysis. Statistical significance was set at a *p*-value of <0.05.

## 3. Results

### 3.1. Characteristics of the Study Population

Seventy-three WLWH with a mean age of 43 ± 9 years (ranging from 25 to 60 years) had eligible BMD data and were included in this analysis (Table 1). The majority (44%) of the women were Caucasian, 25% defined themselves as Aboriginal, and 16% were African. The vast majority of WLWH had some form of cART exposure; only two participants (3%) were cART-naïve. The majority were well treated with most (90%) having CD4 counts over 200 cells/µL, and 66% havingan undetectable HIV plasma viral load (<40 copies/mL). Eighty percent had HIV viral loads below 250 copies/mL. Based on a positive HCV PCR, 23% had active HCV co-infection at the time of the clinic visit.

The 280 women aged 25–60 years in the local CaM*os* cohort were randomly selected, within that same age range, from the local population. Their data are shown in Table 1 and indicate that the groups were similar in age but that the WLWH group had a lower percentage of Caucasian and East Asian women and a higher proportion of African-Canadian and Aboriginal women. Body mass index was similar, as was the number of live births. Bone mineral density values overall were similar between the two cohorts but women in CaM*os* tended to have higher spine and total hip mean BMD values.

### 3.2. Comparison of the Lumbar Spine (LS), Total Hip (TH), and Femoral Neck (FN) BMD of Women Living with HIV to a Reference Population of Women (CaMos)

**Figure 1:**
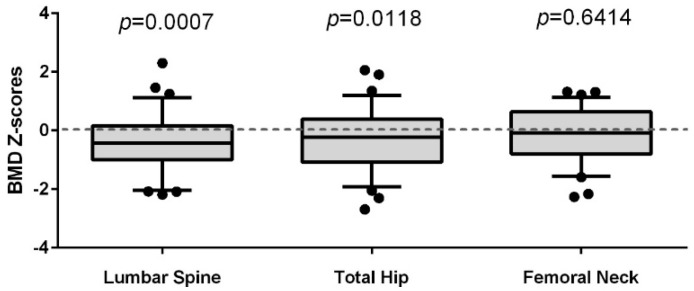
Standard deviations (*Z*-scores), matched by decade of age, of the bone mineral density (BMD) at the lumbar spine, total hip, and femoral neck in WLWH (*n* = 56), related to BMD in a regional randomly selected population of women (*n* = 290). Women included in the analysis were between 25 and 50 years of age. One sample *t*-test was used to determine if the means deviated significantly from population controls.

To assess how the BMD of our cohort of WLWH compared to a CaM*os* reference sample of unselected women from the local population, we calculated *Z*-scores by decade of age. WLWH showed the greatest relative deficit at the LS BMD site, having a mean *Z*-score difference of −0.39, 95% CI [−0.61, −0.17], *p* < 0.001. TH BMD was also reduced, having a mean *Z*-score difference of −0.29, 95% CI [−0.52, −0.07], *p* = 0.012 (Figure 1). FN BMD was not significantly different, and there was also no difference in the average body mass index (BMI) between the groups (*p* = 0.641).

**Figure 2:**
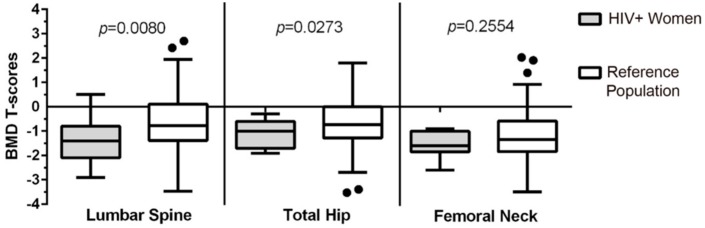
*T*-Scores for the bone mineral density (BMD) at the lumbar spine, total hip, and femoral neck in WLWH aged ≥50 years of age (*n* = 17) and a reference population of unselected women in the Canadian Multicentre Osteoporosis Study from same geographical region (*n* = 167) between 50 and 60 years of age.

A similar outcome was observed when only looking at BMD *T*-Scores of women 50 years of age and older; WLWH (*n* = 17) had a mean difference of −0.74, 95% CI [−1.34, −0.15], *p* = 0.015 at the LS and −0.52, 95% CI [−1.01, −0.02], *p* = 0.043 at the TH (Figure 2). Again, FN BMD values were not different between WLWH and age-similar CaM*os* controls*.*

### 3.3. Predictors of BMD in Women Living with HIV

We investigated factors related to BMD at the LS, TH, and FN in WLWH using linear regression modeling. In univariate analyses, we first assessed the contribution of cART exposure (months of therapy), specifically looking at protease inhibitor (PI) exposure, tenofovir (TDF) exposure, HCV co-infection, CD4 counts, current HIV viral load, age, LTL, BMI, smoking, and parity to BMD at the three sites. LTL (R^2^ = 0.09, *p* = 0.009) and BMI (R^2^ = 0.06, *p* = 0.042) were the strongest univariate predictors of LS BMD. When entered into a multivariate regression model, LTL remained the greatest independent predictor variable for LS BMD (Table 2). For both TH and FN BMD, cumulative months on cART, age, and BMI were significant univariate predictors. Due to the high collinearity of cART and age, only BMI and age remained significantly associated with lower BMD at these two hip sites in the final model (Table 2).

### 3.4. Predictors of LTL in Women Living with HIV

The only variable showing a significant association with LTL was lifetime tenofovir (TDF) exposure, F (1, 71) = 4.384, *p* = 0.04, R^2^ = 0.058 (slope = −11.53 ± 5.5). The median length of time of TDF exposure was 23 months (range 0–85). Fifty-nine (81%) of the 73 WLWH had been exposed to TDF. The mean age of TDF-exposed women was 42.7 ± 8 years; their mean LTL was 2.88 ± 0.5. The mean age of TDF naive women (*n* = 14) was 44.4 ± 10 years; their mean LTL was 2.94 ± 0.5. This relationship between TDF exposure and LTL may account for the loss of the expected inverse relationship between age and LTL, and the observation that age failed to contribute significantly to LTL in this cohort.

## 4. Discussion

The results of this study support the perspective that HIV infection and its treatment are related to accelerated bone aging [19]. Compared to the CaM*os* local reference population of similarly aged women, WLWH had lower BMD, particularly the LS BMD, although it is within the population normal range. LS BMD includes at least 50% trabecular (or cancellous) bone. This relative reduction in spinal BMD was independent of age and was most strongly linked to shorter LTL. Conversely, FN BMD, with its greater cortical bone component [35], was not significantly different between controls and WLWH. Trabecular bone has an accelerated metabolism and responds more rapidly to bone loss and turnover than cortical bone [36,37]. This may explain the observation that the most pronounced differences in BMD were seen in the LS of WLWH in this age group, most of whom would be expected to be either pre- or perimenopausal. WLWH in this study also had relatively reduced TH BMD compared to the regional reference cohort of women. The TH has an intermediate composition of trabecular and cortical bone, and TH BMD was primarily dependent on age and BMI.

The link between shorter LTL, usually taken as a measure of cellular senescence [38], and osteoporosis has been previously shown in a general population of 2150 randomly selected women [24]. Shorter LTL in PLWH compared to HIV-negative controls has been previously observed [26,39]. This is the first demonstration that shortening of LTL is associated with reduced LS BMD in WLWH. Interestingly, a recent study found telomere length was shorter in osteogenic precursor cells in peripheral blood in HIV-positive men between 20 and 25 years of age, particularly those who were infected perinatally and had lower BMD values [40]. However, the bone site most affected in this relatively young cohort of HIV-positive men was not the LS, but rather the distal radius, followed by the FN and TH [40], suggesting that there may be gender differences between HIV-positive men and women with respect to the type and location of bone loss.

Both HIV status and cART use can contribute to cellular senescence of bone marrow-derived mesenchymal stem cells, which serve as osteoprogenitor cells and are important for bone formation and remodeling [41,42,43]. Of our available data, the strongest link to LTL was exposure to TDF, an increasingly common component of cART worldwide, known to affect bone density, which overpowered the expected relationship between LTL and age [44]. This is noteworthy, as it was demonstrated that TDF exhibits the most potent inhibition of telomerase activity and the greatest telomere shortening of five nucleotide reverse transcriptase inhibitors tested at physiological concentrations in vitro [45]. Our findings suggest one consequence of this drug’s effect relates to compromised bone health in WLWH.

Many studies have now linked cART to low BMD in PLWH, and there is a general consensus that a net bone loss of between 2% and 6% can be expected within the first 2 years of cART initiation, depending on the pharmacological combination [1,46,47]. The majority of the available data, however, included relatively short periods of follow-up (most studies being only 2 years long) and overwhelmingly these data report studies in men.

An important question is whether lower BMD translates clinically into an increased risk of fractures in WLWH. This was answered by some of the studies mentioned above, as well as by Triant and colleagues [48]. In that population-based study, conducted between October 1996 and March 2008, there was a comparison of fracture prevalence in HIV-positive and HIV-negative patients in the USA that involved 8525 HIV+ and 2,208,792 HIV-negative patients. Similar to our findings [6], the authors presented an increased risk of fractures in WLWH compared to controls [48]. Among women, the overall fracture prevalence was 2.49 vs. 1.72 per 100 persons in PLWH vs. HIV-negative patients (*p* = 0.002). WLWH had a higher prevalence of vertebral fractures (0.81 vs. 0.45; *p* = 0.01), but had a similar prevalence of hip fractures as their HIV-negative counterparts (0.47 vs. 0.56; *p* = 0.53) [48]. However, fracture risk was elevated *starting at age 30* (i.e., in both pre- and menopausal WLWH). This suggests that to prevent future fractures awareness and prevention of osteopenia/osteoporosis needs to start very early for women living with HIV.

Our study was aimed at addressing some of the gender gaps by providing further data related to how LS, TH, and FN BMD of women living with HIV compare with the regional reference population, and what factors associated with their relatively poor bone health. It is a strength of this study that it compared local WLWH with a population-based regional CaM*os* control cohort and that, for the first time, we showed a relationship between the aging-related variable, leukocyte telomere length, and BMD in WLWH. This study is limited, however, by the retrospective nature of data obtained in these WLWH, by the cross-sectional design, and by the fact that more African-Canadian and Aboriginal women were in the HIV-positive group compared with the CaM*os* cohort, thus were were unable to adjust for ethnicity in our standardized BMD assessment in the younger women. It is also limited by lacking a control group that was similar in ethnicity and lifestyle to the WLWH.

## 5. Conclusions

Bone health is compromised in WLWH, even in those remaining premenopausal. Spinal BMD, which has a significant trabecular bone component, is notably lower, especially in WLWH over age 50, independent of age. Reduction of leukocyte telomere length (LTL) was strongly associated with reduced LS BMD, and this may relate to the role of cellular senescence in the loss of bone renewal potential—especially in the context of specific cART therapies, such as tenofovir, which may further accelerate telomere shortening. Guidelines for the care of WLWH need to take this new information into consideration.

Further research, awareness, and new strategies for maintaining and improving the strength and health of women’s bones are needed, and should begin the day a woman is diagnosed with HIV. Only then will we be able to better prevent fragility fractures in WLWH as they age.

## Figures and Tables

**Table 1:** Demographics and mean areal bone mineral density characteristics of Women living with HIV and women from the adult cohort of British Columbia Canadian Multicentre Osteoporosis Study (CaM*os*). Mean ± SD, median [range], or *n* (%) are reported. WLWH = women living with HIV, BMI = body mass index, DXA = dual energy X-ray absorptiometry.

Demographics	WLWH (*n* = 73)	BC CaM*os* Cohort (*n* = 280)
Mean age at DXA (years)	43 ± 8.7	50 ± 8.1
Mean age of women <50 years	39 ± 6.3	42 ± 6.5
Mean age of ≥50 years	55 ± 2.6	55 ± 3.1
Mean BMI (kg/m2) at DXA	25.4 ± 6.4	26.0 ± 5.1
Height (cm)	162.5 ± 7.3	161.4 ± 6.3
Weight (kg)	67.0 ± 17.2	68.3 ± 14.2
Median number of live births	2 [0–6]	2 [0–7]
Mean smoking pack years	8.6 ± 12.5	^
Mean relative leukocyte telomere length	2.88 ± 0.52	─ *
Race/Ethnicity		
Caucasian	32 (44%)	224 (80%)
Aboriginal	18 (25%)	1 (<1%)
African-Canadian	12 (16%)	0
South Asian	5 (7%)	16 (6%)
Asian	3 (4%)	39 (14%)
Other	3 (4.0%)	0
History of illicit drug use		
Yes	19 (26%)	—
No	43 (59%)	—
Unknown	11 (15%)	—
Combination antiretroviral therapy		
Naïve	2 (3%)	—
Experienced	71 (97%)	—
Mean lifetime protease inhibitor use (months)	47 ± 43	—
Mean lifetime tenofovir use (months)	28 ± 25	—
CD4 count at visit (cells/µL)		
≤200	7 (10%)	—
>200	65 (90%)	—
HIV plasma viral load at visit (copies/mL)		
<250	57 (80%)	—
≥250	14 (20%)	—
Active HCV co-infection		
Yes	17 (23%)	—
No	56 (77%)	—
Bone mineral density (g/cm^2^)		
Lumbar Spine (L1–4)	0.97 ± 0.1	0.99 ± 0.1
Femoral Neck	0.77 ± 0.1	0.76 ± 0.1
Total Hip	0.89 ± 0.1	0.91 ± 0.1

* These HIV-specific data were not obtained in the CaM*os* population-based control cohort; ^ Lifetime 20-cigarette packets smoked (*n* = 129) = 5303 ± 5570. Regrettably, we do not have the cigarette data recorded as pack-years for CaM*os*.

**Table 2:** Multiple linear regression models predicting bone mineral density (BMD) of the lumbar spine (LS), total hip (TH), and femoral neck (FN) of all WLWH (ages 25–60; *n* = 73).

	Standardized β	95% CI for β	*p*-Value
**LS BMD Overall Model: R^2^ = 0.148; F (2, 70) = 6.068, *p* = 0.004**			
Leukocyte telomere length	0.301	0.019, 0.125	0.008
BMI (kg/m^2^)	0.237	0.000, 0.009	0.035
**TH BMD overall model: R^2^ = 0.273; F (2, 70) = 13.128, *p* < 0.001**			
BMI (kg/m^2^)	0.350	0.003, 0.011	0.001
Age (years)	−0.317	−0.007, −0.002	0.003
**FN BMD overall model: R^2^ = 0.248; F (2, 70) = 11.512, *p* < 0.001**			
BMI (kg/m^2^)	0.212	0.000, 0.007	0.049
Age (years)	−0.412	−0.008, −0.002	<0.001
